# Editorial: Chronic pain management and psychological distress in older adults

**DOI:** 10.3389/fmed.2023.1201361

**Published:** 2023-06-22

**Authors:** Simone Scarlata, Damiana Mancini

**Affiliations:** ^1^Fondazione Policlinico Universitario Campus Bio-Medico, Rome, Italy; ^2^Research Unit of Internal Medicine, Department of Medicine and Surgery, Università Campus Bio-Medico di Roma, Rome, Italy; ^3^Amaltea Private Practice, Rome, Italy

**Keywords:** chronic pain management, psychological distress, older adults, non-communicable chronic diseases, olistic point of view

The prevalence of chronic pain increases with age and it was found that about 30.8% of adults aged ≥65 experience chronic pain (1). The US initiative *National Pain Strategy* uses the term “High Impact Chronic Pain (HICP)” for people suffering from pain so chronic that it substantially impacts their life (work/social/self-care areas) for a minimum period of 6 months. Since chronic pain translates to relevant financial, physical and emotional burdens on families (and on society in general), finding treatments is crucial to patients who see their life being limited by chronic pain.

Moreover, it was reported that chronic pain plays a role in the risk of early death, with an increased risk of 57% for excess all-cause mortality (2).

Surprisingly, there are not many studies focussing on chronic pain in the aging population (≥65 years) and therefore the evidence supporting the role of pharmacologic or non-pharmacologic intervention is scarce (3). It then becomes crucial to focus on treatments for symptoms responsible for disability and a diminished quality of life.

As Boakye et al. pointed out, “pain has both sensory and emotional-affective components. The integration and perception of these components provides an aversive signal that can protect an organism from potential or additional tissue damage (“adaptive” or “good” pain). […] “Disruptive” or “bad” pain can arise when pain persists beyond its biologically useful function, and becomes chronic in nature” (4).

We know that the association between chronic pain and depression has been extensively demonstrated. Noteworthy, when a relevant mood change is occurring this is often linked to an alteration in tolerance, perception and threshold to pain. Boakye et al. state that “a significant proportion of persons with a pain condition also has a higher level of depression or anxiety, and the severity of pain expressed by depressed persons is predictive of the time required for remission of the depressive illness after treatment” (5). We can therefore infer that depression reduces the pain threshold, but also that pain can lead to a depressive condition. It is then important for a clinician to better understand the intricacies and dependencies of pain and depression, in order to help this type of patients in a better way.

The cited study continues by observing that “pain is a major obstacle to achieving full remission during the treatment of depression in ~70% of depressed people who responded to treatment, those who were also experiencing some forms of pain were less likely to have a total remission of their physical and emotional issues, and as such continued to have residual symptoms.”

As evidence shows, pain and depression can coexist and have almost the same neurobiological processes. For example, as Chang et al. (6) continue: “Among people with a chronic pain diagnosis, sleep disturbance is a common clinical symptom, with 50%−90% of people with pain reporting sleep disturbances of some description, with insomnia being the most common problem.” This may also be due to the difficulty of falling asleep, and remain asleep, when chronic pain is present (7). What emerges from these studies then, is an interconnection among chronic pain, depression and insomnia. Understanding these interactions may lead us to more effective treatment strategies.

When treating chronic pain in aging people with pharmaceutical products, the results are not completely satisfactory and are often limited by side effects, such as: urinary retention, constipation, sedation, cognitive impairment, and increased risk of falls. Unfortunately, the impact of polypharmacy, frailty and side effect is not known because this population is not adequately represented in clinical trials for chronic pain treatments (8). For this reason, much more insight on the pharmacokinetic and pharmacodynamic of antalgic drugs is needed, as well as a deeper understanding of the hazard related to polypharmacy. A more comprehensive knowledge over the long-term consequences of pain killer use in subjects over 80 years old, would also be urgent.

On the other hand, non-pharmacologic therapies for pain management and psychological distress relief have been around for a long time. They include among the many: non-invasive brain and transcutaneous electrical nerve stimulation techniques, cognitive behavioral programmes or techniques of self-hypnosis, acupuncture, psycho-therapies administrated even remotely through the internet (9).

Also, musculoskeletal pain is often treated with the help of alternative medicine, reported to be of some effect by 59%−90% of patients. It seems then we could avail of alternative therapies in the treatment of chronic pain, however the evidence based on such therapies is inconclusive, due to a sever lack of: methodological standardization, high quality clinical trials, and data on short and long-term efficacy. Some therapies appear to be effective on specific types of pain, although there is evidence refuting such claims. To date, we cannot conclude that a non-pharmacological approach alone may replace pharmaceutical treatment. However, using alternative medicine as adjuvant strategies may reduce the need of opioids for the patient.

The chosen articles composing this monographic issue of Frontiers in Medicine, deal with hot topics such as: the lack of representation of elderly patients in randomized clinical trials, a factor which forces a complicated, perhaps risky translation of posology and therapeutic indications between different and not completely comparable study populations (Krysa et al.).

It also discusses the determinants of psychological distress among subjects suffering from chronic pain in contexts alternative to the prevailing ones, such as rural contexts (Yang et al.). Also, it clarifies the factors associated with chronic pain and psychological distress in different pathological conditions, both neoplastic (Zhai et al.) and non-neoplastic (Gower et al.) with potential interventions. Furthermore, it proposes integrated approach models for the management of institutionalized patients suffering from chronic pain (Tse et al.). And finally, it introduces the topic of integrative, non-pharmacological approaches, coming from traditional medicine (Guo et al.) or associated with manual therapy (Wu et al.).

Even if the articles we refer to may prompt more questions than answers, they may help assessing the condition of our literature at present, where the need for evidence on the management of chronic pain and the associated psychological distress in aging people is rather urgent, so that healthcare providers, patients and policy makers can act accordingly.

Like Busse et al., we think that researchers should cooperate with patients living with chronic pain and include them in their teams in order to explore the most relevant priorities and design the study (10). The experiences and perspectives of such patients/team members can offer invaluable insights. As the researches state: “symptom relief is critical, but so are physical functioning, sleep quality, financial security, social integration, employment and other meaningful activity, and adverse events. Many clinical trials of chronic pain fail to report outcomes of interest to those who live with chronic pain, which greatly limits their utility” (11).

The current Research Topic of Frontiers of Medicine—Geriatrics is, therefore, a call to action.

## Author contributions

All authors drafted the work and revised it critically for important intellectual content and provided a final approval of the version to be published.
